# Confidence of Emergency Department doctors in managing ophthalmic emergencies: a systematic review

**DOI:** 10.1038/s41433-024-03115-z

**Published:** 2024-05-10

**Authors:** Jessica Mendall, Abraham Tolley, Veronica Parisi, Stella Hornby, Ruth Brown, Victoria Nowak

**Affiliations:** 1 University College London, London, UK; 2 University of Cambridge School of Clinical Medicine, Cambridge, UK; 3 University College London Library Services, London, UK; 4 Oxford Eye Hospital, Oxford University Hospitals NHS Foundation Trust, Oxford, UK; 5 Imperial College Healthcare NHS Trust, London, UK; 6 The National Hospital for Neurology and Neurosurgery, London, UK

**Keywords:** Epidemiology, Education, Eye diseases, Epidemiology, Health services

## Abstract

**Background:**

Eye emergencies constitute a significant portion of attendances to general Emergency Departments (EDs) in the UK, therefore it is important to assess the confidence of doctors who work in this setting in managing these potentially sight- and life-threatening presentations. This systematic review aims to assess the confidence of UK doctors working in general EDs in managing ophthalmic emergencies.

**Methods:**

MEDLINE (Ovid), EMBASE (Ovid), ProQuest Central and Web of Science databases and grey literature were searched from inception to 1 October 2022 for publications that (1) featured doctors working in UK general EDs, (2) assessed doctors’ confidence in managing ophthalmic emergencies, (3) contained original data, (4) were full-text, and (5) written in English. Methodological quality was assessed using the AXIS tool.

**Results:**

462 articles were screened, and 7 papers included for data extraction, which collectively assessed the confidence of 956 doctors working in EDs in managing ophthalmic emergencies. There was a widespread lack of confidence amongst foundation doctors, which has worsened over time. Most doctors lacked confidence in performing funduscopy and using the slit-lamp, and considered formal ophthalmology training received in EDs to be inadequate.

**Conclusions:**

Evidence suggests a lack of confidence amongst foundation doctors in managing ophthalmic emergencies. High-quality evidence investigating the confidence amongst more experienced Emergency Medicine (EM) physicians was lacking. It is important to assess why foundation doctors feel so ill-prepared to manage eye emergencies and develop further ophthalmic training for doctors working in EDs. Further investigation exploring the confidence of EM trainees and consultants is required.

## Introduction

In the United Kingdom, Emergency Eye Care (EEC) services are provided by both dedicated ophthalmic Emergency Departments (EDs) and general EDs [1]. Eye emergencies comprise up to 6% of all attendances to general EDs in the UK [2, 3]. The demand for EEC is growing, with annual attendances at Moorfields Eye Hospital and the Western Eye Hospital, London’s largest ophthalmic EDs, increasing by 7.9% and 9.6% per year respectively [4].

Many acute eye presentations are sight- or life-threatening, requiring immediate attention and early treatment to prevent a poor prognosis. The most common ophthalmic presentations to the ED include trauma (mainly corneal or conjunctival abrasions or foreign bodies), inflammatory disease (largely conjunctivitis and blepharitis), subconjunctival haemorrhage and styes [3, 5]. Rarer but high-risk emergencies also present to general EDs, including acute glaucoma, endophthalmitis, retinal artery occlusion and retrobulbar haemorrhage (RBH) [2]. In 2017, up to 20 patients per month in the UK were suffering preventable permanent visual loss due to delays in treatment for which the health service was responsible [6]. It is therefore important that doctors working in general EDs can confidently and accurately manage acute ophthalmic presentations or recognise that specialist ophthalmic input is required, in order to ensure the provision of timely and high-quality patient care.

Doctors working in general EDs vary in experience, from newly-qualified foundation doctors undertaking four-month rotations, to Emergency Medicine (EM) trainees, specialty and associate specialist (SAS) doctors and EM Consultants. Whilst foundation doctors would not be expected to independently manage ophthalmic emergencies without senior support, it is expected that by the end of their training, EM physicians can diagnose and manage red and painful eyes, manage eye trauma including foreign body removal and perform lateral canthotomy for RBH [7]. Evidence suggests many foundation doctors working in general EDs have received little ophthalmic training [8, 9] and lack confidence in diagnosing and managing ophthalmic conditions [10]. The confidence levels amongst other ED doctors including EM registrars, consultants and SAS doctors, and for managing specific ophthalmic emergencies, are not clear.

Examination using the direct ophthalmoscope and slit lamp are essential in the diagnosis of many ophthalmic emergencies. Despite being readily-available, an increasing number of medical students and doctors are not confident in using the direct ophthalmoscope [11]. Additionally, whilst slit lamps are available in 79.8% of UK EDs, there is a lack of formal slit lamp training for junior doctors in the ED [10].

To our best knowledge, no systematic review assessing the confidence of doctors working in general EDs in managing ophthalmic emergencies currently exists. In this systematic review, we aimed to collect and evaluate the literature assessing the confidence of doctors working in general EDs in managing ophthalmic emergencies in the UK for the first time, stratifying by training grade where possible and identifying where future research is required. As secondary outcomes, we also explore the confidence of these doctors in performing funduscopy and using the slit lamp, and training received in the ED.

## Methods

The review protocol was registered on the PROSPERO international prospective register of systematic reviews (registration number: CRD42022365160). The Preferred Reporting Items for Systematic Reviews and Meta-Analyses (PRISMA) checklist was followed in the preparation of this manuscript. The only amendments to the original protocol registered on PROSPERO were the addition of two further exclusion criteria: “not full text” and “not containing original data”.

### Search methods

Expert opinion from a University College London librarian (VP) was sought to develop the search strategy. MEDLINE (Ovid), EMBASE (Ovid), ProQuest Central and Web of Science were searched from inception to 1st October 2022, using a combination of search terms relating to (1) doctors, (2) emergency departments, (3) eye emergencies, and (4) confidence (Supplementary Table 1). Forward and backward citation searching of relevant publications was conducted using Google Scholar and Web of Science. The following grey literature were also searched using the search terms “doctor AND eye AND emergency”: ProQuest Dissertations & Theses Global; EThOS; The King’s Fund; and Open Grey. Google Scholar was searched for publications that included the terms “doctor AND eye” in their title.

### Screening and selection

Titles and abstracts of search results from the databases were uploaded to EndNote, where duplicates were removed. Titles and abstracts of identified citations were subsequently screened using Rayyan. Full texts of potentially relevant papers were studied. Publications included: (1) featured doctors working in general EDs in the UK; (2) assessed the confidence of doctors in managing ophthalmic emergencies; (3) contained original data; (4) were full-text; and (5) were written in English. Publications excluded: (1) did not feature doctors working in general EDs in the UK; (2) did not assess the confidence of doctors in managing ophthalmic emergencies; (3) did not contain original data; (4) had no full-text; and (5) were not written in English. Screening was independently and blindly conducted by two reviewers (JM and AT) at both stages, and any disagreements were resolved by discussion.

### Data extraction

One reviewer (JM) extracted and tabulated the following data from eligible articles: (1) study identification (author details, year of publication); (2) study group characteristics (training grade of doctors, sample size); (3) type of ophthalmic emergency assessed (if specified); (4) methods (study design, how confidence was measured); and (5) key results. The second reviewer (AT) independently verified the extracted data.

### Data synthesis

Meta-analysis was not performed due to methodological heterogeneity in the included studies. Data were synthesised and analysed narratively.

### Appraisal of evidence

Methodological quality of included studies was assessed independently by two reviewers (JM and AT) using the Appraisal tool for Cross-Sectional Studies (AXIS) [12], with any disagreements resolved by discussion. All papers were included in the review, since no cut-off score to determine whether studies are of sufficient quality was stipulated in the final AXIS tool [12]. However, this systematic review’s discussion is weighted towards papers with a stronger AXIS score. The AXIS scores were used to identify weaknesses in study design and inform recommendations for future surveys.

## Results

### Study selection

**Fig. 1:**
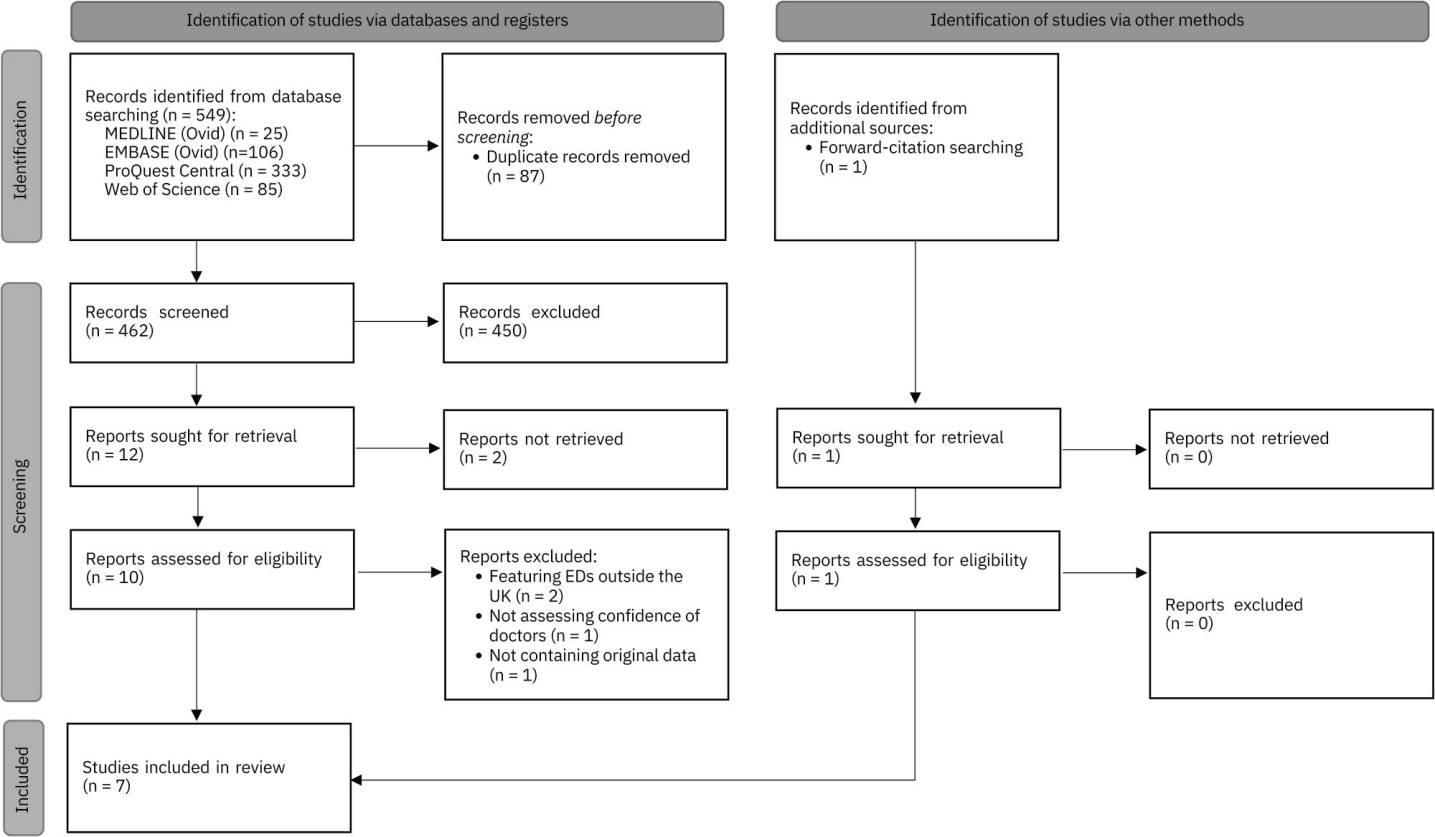
PRISMA diagram. PRISMA flow chart of search and selection process.

549 publications were retrieved from electronic library databases, of which 87 were duplicates (Fig. 1). 450 papers were excluded on the basis of their title and abstract because they did not feature doctors working in EDs in the UK, did not assess the confidence of doctors in managing ophthalmic emergencies or did not present original data. Two further papers were excluded because no full-text was provided and neither included author contact details. Full text screening of the remaining ten papers led to the exclusion of a further four [13–16] (with the reasons for exclusion provided in Supplementary Table 2). One additional eligible paper was identified through forward-citation searching of the included papers. In total, seven studies were included in the systematic review, all cross-sectional in design.

### Study characteristics

The characteristics of the seven included studies are summarised in Table 1 [10, 17–22]. They collectively assessed the confidence of 956 ED doctors (721 Foundation doctors/Senior House Officers (SHOs), 117 EM Specialty Trainees (ST1-8), 21 SAS doctors and 97 Consultants) in managing ophthalmic emergencies. Five studies explored the confidence of SHOs or Foundation Year 2 (FY2) doctors only in managing ophthalmic emergencies, and the other two included doctors of all training grades. Five studies focussed on general ophthalmic emergencies [10, 17, 19] or general ophthalmic presentations (such as the “red eye” and “blurred vision”) [20, 21], and the other two studies assessed specific ophthalmic emergencies: corneal abrasion [18] and RBH [22].

**Table 1:** Summary of included studies.

Author (year)	Training grade of doctors	Population size, *n*	Study location	Type of ophthalmic emergency assessed	Study design	AXIS score
Tan et al. (1997) [17]	SHOs	192	EDs across the UK	General ophthalmic emergencies	Telephone survey conducted with 1 randomly-chosen SHO from each ED in the UK that responded (192/226 EDs responded, response rate 84.9%)	13
Thyagarajan et al. (2006) [18]	SHOs	9	King’s College Hospital, London	Corneal abrasions	Survey of SHOs before and after the introduction of guidelines for management of corneal abrasions	8
Sim et al. (2008) [19]	SHOs	133	EDs across the UK	General ophthalmic emergencies	Telephone survey conducted with 1 randomly-chosen SHO from each ED in the UK that responded (133/167 EDs responded, response rate 79.2%)	16
Saifuddin & Brookes (2014) [20]	FY2s	21	King’s College Hospital, London	“red eye” and “visual disturbance”	Survey of FY2 doctors in the local ED	10
Murray et al. (2016) [21]	All training grades from FY1 to Consultant (12 FY1/2, 19 CT1/ST2, 12 ST3-6 + , 13 SAS, 9 Consultant)	65	West Midlands	Red eye, acute loss of vision, ocular trauma and chemical injury	Survey of doctors working in EDs in 6 NHS Trusts in the West Midlands	8
Edmunds et al. (2019) [22]	All training grades from FY1 to Consultant (8 FY1/2, 55 ST1-4, 31 ST5-8, 8 SAS doctors, 88 Consultant)	190	Birmingham, Bristol, Cardiff, Dundee, Glasgow, London and Plymouth	Retrobulbar haemorrhage (RBH)	Online survey of doctors working in EDs in 7 UK locations	14
Sim et al. (2020) [10]	FY2s	346	EDs across the UK	General ophthalmic emergencies	Combined online and telephone survey of FY2s in each ED in the UK that responded (202/210 EDs responded, response rate 96.2%)	18

*SHO* Senior House Officer, *FY1* Foundation Year 1, *FY2* Foundation Year 2, *CT1* Core Trainee year 1, *ST* Specialty Trainee, *SAS* Specialty and Associate Specialist.

### Quality assessment

The AXIS scores (out of 20) of included studies are summarised in Table 1, with the detailed risk of bias assessment available in Supplementary Table 3. Studies were of variable quality, with a mean AXIS score of 12/20. Common methodological weaknesses were lack of statistical analysis, failure to address non-response bias, inadequate information on non-responders and failure to mention what ethical approval or consent of participants was sought. No studies were excluded as no cut-off value for the AXIS score exists [12], so the discussion is weighted towards the higher-quality papers.

### Confidence of doctors working in general EDs in managing ophthalmic emergencies

Five studies assessed the confidence of SHOs or FY2s only in managing ophthalmic emergencies [10, 17–20], whilst two studies assessed the confidence of both foundation doctors and EM physicians (including ST1-8 EM Specialty Trainees, EM consultants and SAS doctors) [21, 22] as shown in Table 2. Overall, confidence of FY2s and SHOs in managing ophthalmic emergencies was lacking. Three national surveys that used a similar questionnaire, conducted in 1993 [17], 2003 [19] and 2018 [10], demonstrated a decline in confidence amongst SHOs or FY2s in managing ophthalmic emergencies over time. In 1993 and 2003, 31.3% (60/192) and 36.1% (48/133) of SHOs respectively felt “confident” in managing ophthalmic emergencies; this dropped to 2.3% (8/346) of FY2s feeling “confident” in 2018. In a 2014 paper, confidence amongst 21 FY2 doctors averaged at 2.2/5 and 2.3/5 for managing “visual disturbance” and the “red eye” respectively [20].

**Table 2:** Confidence of doctors working in general EDs in managing ophthalmic emergencies.

Author (year)	Training grade of doctors assessed	Population size, *n*	Type of ophthalmic emergency assessed	How was confidence measured?	Summary of results
Tan et al. (1997) [17]	SHOs	192	General ophthalmic emergencies	(a) “confident”, (b) “a little confident” or (c) “not confident”	31.3% (60/192) “confident”, 65.1% (125/192) “a little confident”, 3.6% (7/192) “not confident”
Thyagarajan et al. (2006) [18]	SHOs	9	Corneal abrasions	(a) “confident”, (b) “a little confident” or (c) “not confident”	10% (1/9) “confident”, 90% (8/9) “a little confident” or “not confident” pre-guideline introduction. Post-guideline introduction, 100% (6/6) “confident”.
Sim et al. (2008) [19]	SHOs	133	General ophthalmic emergencies	(a) “confident”, (b) “a little confident”, or (c) “not confident at all”	36.1% (48/133) “confident”, 55.6% (74/133) “a little confident”, 8.3% (11/133) “not confident”
Saifuddin & Brookes (2014) [20]	FY2s	21	“red eye” and “visual disturbance”	On a scale from 0 (low confidence) to 5 (high confidence)	Confidence averaged 2.3/5 for dealing with the “red eye”, and 2.2/5 for dealing with “visual disturbance”
Murray et al. (2016) [21]	All training grades from FY1 to Consultant (12 FY1/2, 19 CT1/ST2, 12 ST3-6+, 13 SAS, 9 Consultant)	65	Red eye, acute loss of vision, ocular trauma and chemical injury	Not enough information provided; options included “confident” and “very confident”	40% (26/65) were “confident” or “very confident” in formulating a management plan for the red eye; 34% (22/65) for acute loss of vision; 32% (21/65) for ocular trauma; and 43% (28/65) for chemical injury
Edmunds et al. (2019) [22]	All training grades from FY1 to Consultant (8 FY1/2, 55 ST1-4, 31 ST5-8, 8 SAS doctors, 88 Consultant)	190	Retrobulbar haemorrhage (RBH)	“Would you be happy performing LC/C [lateral canthotomy and cantholysis] for RBH?” (yes/no)	37.1% (70/190) “yes”, 62.9% (120/190) “no”
Sim et al. (2020) [10]	FY2s	346	General ophthalmic emergencies	Out of (a) “confident”, (b) “a little confident”, or (c) “not confident at all”	2.3% (8/346) “confident”, 38.7% (134/346) “a little confident”, 59.0% (204/346) “not confident at all”

*SHO* Senior House Officer, *FY1* Foundation Year 1, *FY2* Foundation Year 2, *CT1* Core Trainee year 1, *ST* Specialty Trainee, *SAS* Specialty and Associate Specialist.

One small 2006 study assessed the confidence of FY2s in managing a specific ophthalmic emergency: corneal abrasions. 10% of SHOs (1/9) were “confident” in the management and follow-up of corneal abrasions before the introduction of guidelines, which improved to all (6/6) feeling “confident” after guideline introduction [18]. Furthermore, before guideline introduction, 77% (7/9) were confident in the history-taking component but only 22% (2/9) confident in the examination. This improved to all (6/6) becoming confident about history-taking and examination after the guidelines were introduced.

Results from the two studies assessing the confidence of foundation and EM doctors of various grades are difficult to interpret as the breakdown of results were not provided for specific training grades and roles. In a mixed cohort of 65 doctors comprising foundation doctors (FY1 and FY2), EM Specialty Trainees (ST1-8), SAS doctors and consultants, less than half were “confident” or “very confident” in managing ocular trauma (32%), acute loss of vision (34%), the red eye (40%) and chemical injury (43%) [21]. In the study by Edmunds et al. [22], 37.1% (70/190) of doctors, the majority of whom were EM Specialty Trainees (*n* = 86) or consultants (*n* = 88), would be “happy” in performing a lateral canthotomy and cantholysis (LC/C) for RBH [22].

### Confidence of doctors working in general EDs in performing funduscopy and using the slit lamp

Two studies assessed the confidence of ED doctors in performing funduscopy [10, 21], and five studies reported the confidence of ED doctors in using the slit lamp [10, 17–19, 21] (Table 3). 10.7% (37/346) of FY2s in the 2018 national survey felt “confident enough” in performing funduscopy [10]. This figure was slightly higher amongst a mixed cohort of foundation doctors and EM specialty trainees, SAS doctors and consultants, of whom 29% (19/65) “felt competent” in using the ophthalmoscope [21].

**Table 3:** Confidence of doctors working in general EDs in performing funduscopy and using the slit lamp.

Author (year)	Training grade of doctors assessed	Population size, *n*	How was confidence in performing funduscopy measured?	Summary of results: confidence in performing funduscopy	How was confidence in using the slit lamp measured?	Summary of results: confidence in using the slit lamp
Tan et al. (1997) [17]	SHOs	192	Not assessed	N/a	(a) “confident enough”, (b) “a little confident”, or (c) “not confident”	27.6% (53/192) “confident enough”, 27.6% (53/192) “a little confident”, 44.8% (86/192) “not confident”
Thyagarajan et al. (2006) [18]	SHOs	9	Not assessed	N/a	(a) “confident”, (b) “a little confident”, or (c) “not confident”	“Most were not confident about using a slit-lamp”, no further detail provided
Sim et al. (2008) [19]	SHOs	133	Not assessed	N/a	(a) “confident enough”, (b) “a little confident”, or (c) “not confident at all”	39.1% (52/133) “confident enough”, 30.8% (41/133) “a little confident”, 30.1% (40/133) “not confident at all”
Murray et al. (2016) [21]	All training grades from FY1 to Consultant (12 FY1/2, 19 CT1/ST2, 12 ST3-6+, 13 SAS, 9 Consultant)	65	Whether doctors “felt competent” in using the funduscope	71% (46/65) “did not feel competent” in using the funduscope. Only 8% (1/12) of Foundation doctors believed they were competent in performing funduscopy.	Whether doctors “felt competent” in using the slit lamp	68% (44/65) “did not feel competent” in using the slit lamp
Sim et al. (2020) [10]	FY2s	346	(a) “confident enough”, (b) “a little confident”, or (c) “not confident at all”	10.7% (37/346) “confident enough”, 52.3% (181/346) “a little confident”, 37.0% (128/346) “not confident at all”	(a) “confident enough”, (b) “a little confident”, or (c) “not confident at all”	9.8% (34/346) “confident enough”, 25.7% (89/346) “a little confident”, 64.5% (223/346) “not confident at all”

*SHO* Senior House Officer, *FY1* Foundation Year 1, *FY2* Foundation Year 2, *CT1* Core Trainee year 1, *ST* Specialty Trainee, *SAS* Specialty and Associate Specialist.

Confidence in using the slit lamp has fallen amongst SHOs/FY2s over time. In the national surveys of 1993 [17] and 2003 [19], 27.6% (53/192) and 39.1% (52/133) of SHOs felt “confident enough” in using the slit lamp, respectively. Confidence had declined by 2018, where only 9.8% (34/346) of FY2s felt “confident enough” [10]. Additionally, in the 2016 study, 32% (21/65) of the mixed cohort of doctors “felt competent” in using the slit lamp [21]. Whilst this study did not provide a breakdown in the confidence levels for doctors of different training grades, only 12/65 of the participants were FY1/2s, indicating that low confidence was not limited to foundation doctors. One study did not provide sufficient detail to interpret confidence in slit lamp use [21].

### Formal ophthalmology training received in the ED

Five studies assessed whether doctors working in general EDs had received formal ophthalmology training in the ED [10, 17–19, 21], although one study did not report these results [18] (Table 4). In the 1993 [17] and 2003 [19] national surveys, 26.0% (50/192) and 22.6% (30/133) of SHOs reported receiving no formal ophthalmology training in the ED, respectively. More recently, the majority of ED doctors did not receive formal ophthalmology training: in 2016, 63% (41/65) of doctors across a variety of training grades reported receiving “none” [21]. In the 2018 national survey of FY2s, 59.0% (204/346) had received no training, while 54.3% (188/346) recognised that “formal training and teaching” would improve their confidence in managing eye emergencies [10]. In terms of managing RBH, 92.2% (175/190) of doctors across various training grades felt that more training was required for EM physicians in RBH management and performing LC/C [22].

**Table 4:** Formal ophthalmology training received in the ED.

Author (year)	Training grade of doctors assessed	Population size, *n*	How was formal ophthalmology training received in the ED measured?	Summary of results
Tan et al. (1997) [17]	SHOs	192	(a) “enough for my needs”, (b) “some”, or (c) “none”	18.8% (36/192) “enough for my needs”, 55.2% (106/192) “some”, 26.0% (50/192) “none”
Thyagarajan et al. (2006) [18]	SHOs	9	Whether doctors had received formal training in the management of corneal abrasions specifically. (a) “some”, (b) “none”, or (c) “enough for me”	Not reported
Sim et al. (2008) [19]	SHOs	133	(a) “enough for my needs”, (b) “some”, or (c) “none”	32.3% (43/133) “enough for my needs”, 45.1% (60/133) “some”, 22.6% (30/133) “none”
Murray et al. (2016) [21]	All training grades from FY1 to Consultant (12 FY1/2, 19 CT1/ST2, 12 ST3-6+, 13 SAS, 9 Consultant)	65	Whether doctors had received any formal ophthalmology training within the ED	63% (41/65) had received “none”
Sim et al. (2020) [10]	FY2s	346	(a) “enough for my needs”, (b) “some”, or (c) “none”	7.5% (26/346) “enough for my needs”, 33.5% (116/346) “some”, 59.0% (204/346) “none”

*SHO* Senior House Officer, *FY1* Foundation Year 1, *FY2* Foundation Year 2, *CT1* Core Trainee year 1, *ST* Specialty Trainee, *SAS* Specialty and Associate Specialist.

It was widely felt that ophthalmology teaching at medical school was inadequate preparation for doctors for working in EDs. In the 2014 study, 65% (14/21) of FY2s stated that “undergraduate ophthalmology teaching at medical school had not prepared them well for their ED placement”, and 60% (13/21) felt that “more teaching as junior doctors would be beneficial” [20]. In 2016, 57% (37/65) of doctors of mixed training grades “felt their undergraduate ophthalmology teaching was inadequate” [21]. 92.2% (175/190) of a mixed cohort of doctors ranging from foundation year to EM consultant felt that “more training in LC/C is required for emergency department doctors”.

## Discussion

To our best knowledge, this is the first systematic review to investigate the confidence of UK doctors working in general EDs in managing ophthalmic emergencies. Seven cross-sectional studies were identified, collectively assessing the confidence of 956 doctors across various training grades. We found that confidence has been consistently low amongst SHO/FY2 doctors working in EDs in managing ophthalmic emergencies. The majority of doctors studied were of SHO/FY2 level, and studies assessing the confidence of EM specialty trainees, SAS doctors and consultants were lacking, hence it was difficult to draw conclusions for these doctors. Furthermore, several of the available studies were of poor quality and limited size, with relatively low AXIS scores.

### Confidence of foundation/SHO doctors in managing ophthalmic emergencies

The confidence of foundation/SHO doctors in managing ophthalmic emergencies was assessed in most studies, and found to be generally low. The three national surveys [10, 17, 19], which had relatively high AXIS scores (of 13, 16 and 18), provide strong evidence that confidence is lacking in managing ophthalmic emergencies amongst SHO/FY2 doctors [10, 17, 19]. Despite the shift towards a more competency-based Foundation Programme for junior doctors introduced by the Modernising Medical Careers (MMC) initiative in 2005 [23], the national surveys revealed reductions rather than improvements in the confidence of FY2 ED doctors in managing eye emergencies over time [10]. Why confidence amongst FY2s/SHOs has dropped over time is unclear. This low confidence may stem back to inadequate training and limited exposure to ophthalmology at medical school [24], likely compounded by the lack of formal ophthalmology training provided in the ED. It will be interesting to see the impact of the Medical Licensing Assessment in the UK, starting in 2024-25 [25], the syllabus for which includes a comprehensive list of 12 ophthalmic presentations and 18 ophthalmic conditions.

Similar themes have been highlighted in other specialties. A small single-centre study (*n* = 14) of SHOs working in EDs revealed confidence was particularly low in managing minor injuries [26]. Larger studies have revealed that a majority of foundation and junior doctors lack confidence in diagnosing and managing a range of ENT emergencies including epistaxis, peritonsillar abscess and post-tonsillectomy bleeds [27, 28] and oncological emergencies [29]. This could reflect the duration of ENT teaching in medical school, which like Ophthalmology teaching, is often limited in duration [27, 30].

The implications of FY2/SHO doctors having low confidence in managing ophthalmic emergencies are that their knowledge and certainty in how to appropriately manage ophthalmic emergencies may be lacking [31]. However, as long as FY2/SHO doctors seek senior support from more experienced doctors in the ED team and appropriately refer to ophthalmologists where required, this low confidence is unlikely to negatively impact patient outcomes, and could be preferable to overconfidence which may drive dangerous decision-making [31]. Nevertheless, it is important to investigate why confidence is low amongst foundation doctors in managing ophthalmic emergencies, and how it relates to competence, especially in terms of requesting timely senior reviews, in order to develop relevant further training to fill knowledge gaps.

Despite 97.4% of FY2s having access to an ophthalmoscope within their ED [10], and 80% of general EDs possessing slit lamps, we found that confidence in performing funduscopy and using the slit lamp amongst foundation doctors was lacking. This low confidence has been mirrored amongst final year medical students in the UK [11]. Since the General Medical Council include funduscopy as a core competency of newly qualified doctors [32], we believe further funduscopy training for medical students and foundation doctors is vital. Furthermore, although proficiency in using slit lamps takes time and is not a compulsory aspect of the medical school curriculum [32], we suggest anterior segment examination as part of the work-up of common ophthalmic presentations to the ED is an achievable aim for foundation doctors working in EDs. We also report a more general lack of formal ophthalmology training provided in the ED. Perhaps this is due to lack of time, variable shift patterns, the short duration of the ED job for foundation doctors, or because teaching on ophthalmic complaints is not prioritised. However, we identified a positive attitude towards further training amongst doctors working in EDs, with many believing that more teaching would be beneficial and improve their confidence in managing eye emergencies.

### Confidence of EM doctors in managing ophthalmic emergencies

The confidence of EM doctors in managing ophthalmic emergencies is difficult to reliably assess since the studies assessing more senior EM doctors up to Consultants did not break down outcomes between EM doctors and FY1/2 doctors [21, 22]. However, the study investigating RBH management, of relatively high quality with an AXIS score of 14/20, assessed mostly EM doctors (with only 8/190 participants being FY1/2). It is perhaps unsurprising that less than half of surveyed doctors were confident in performing LC/C, since it is an invasive procedure [33] and RBH is uncommon [34]. Furthermore, similar confidence levels have recently been reported in a US study. Whilst 60.2% (198/329) of surveyed EM physicians felt “comfortable” in determining whether LC/C is required in cases of RBH, 40.3% (133/329) of EM physicians felt “comfortable” in performing LC/C for RBH [35]. Nevertheless, more doctors believed it to be the role of the EM physicians rather than Ophthalmologists to perform LC/C (41.0% compared to 36.8%). Given the rapid and potentially sight-threatening effects of RBH [36] and that lateral canthotomy is included in the Royal College of Emergency Medicine’s 2021 curriculum [7], we believe further training for EM doctors in performing this procedure may be warranted.

Low confidence amongst the more senior EM doctors in managing ophthalmic emergencies could have more serious consequences than for FY2/SHO level doctors, given that the majority of patients seen by FY2s/SHOs will be discussed with a senior. Failure of senior EM doctors to recognise ophthalmic complaints and their severity, know how to instigate appropriate immediate management in A&E, and request specialist ophthalmic input when required, could negatively impact patient outcomes. It is therefore imperative that further high-quality studies assess not only the confidence of FY2/SHO doctors in managing ophthalmic emergencies, but also of EM doctors (including EM trainees, SAS doctors and EM consultants).

Future studies could also assess the confidence and potential role of emergency nurse practitioners (ENPs) to assess and triage patients presenting with ophthalmic emergencies, who represent a more stable workforce than foundation doctors rotating through 4-month jobs. ENPs could also provide valuable mentorship for foundation doctors as they have demonstrated a high standard of diagnostic and management skills, accurate triage of patients [37], and have been more accurate than SHOs in assessing patients, particularly in measuring visual acuity and formulating a provisional diagnosis [9].

## Limitations

This systematic review used a PROSPERO-registered and PRISMA compliant framework to conduct an up-to-date evaluation of the literature assessing the confidence of UK ED doctors in managing ophthalmic emergencies. We must however consider the findings of this systematic review in the context of its limitations. Firstly, there were relatively few eligible studies, which were of variable size and quality. Whilst the AXIS tool does not include a cut-off value to reflect sufficient quality [12], three of the included studies had particularly low AXIS scores [18, 20, 21], thus we give these studies a lower weighting in this discussion. The corneal abrasion audit had a very small sample size (*n* = 9 pre-guideline introduction, and *n* = 6 post-guideline introduction), limiting its external validity. The studies by Murray et al. [21] and Saiffudin and Brookes [20] lacked methodological detail. Saiffudin and Brookes measure confidence on a non-validated 5-point scale, and the rationale for this measure and its meaning are not discussed. Future cross-sectional studies assessing ED doctors’ confidence should therefore use the AXIS tool to inform their study design.

Secondly, whilst high-quality multicentre studies provided strong evidence to suggest that confidence amongst SHO/FY2 doctors is low, it was difficult to draw conclusions to ED doctors of other training grades. Studies assessing ED doctors from FY1 level through to Consultant were limited to one region of the UK [21], had limited numbers of certain training grades [21, 22] and did not provide breakdowns in confidence levels for the different training grades [21, 22]. Furthermore, the amount of prior experience different foundation doctors had in the ED was not highlighted in studies; confidence in managing various emergencies is likely to differ at the start and end of a 4-month ED rotation [38]. Thirdly, quantitative synthesis of results was not possible due to heterogeneity in study design. Future studies could use the same validated questionnaire, for example the questionnaire used in the three national surveys [10, 17, 19], but expanded to include specific ophthalmic emergencies, check knowledge on when to seek senior review, and survey more senior EM doctors. This would enable comparisons to be more accurately drawn between studies and enable quantitative synthesis of results for future reviews.

## Conclusions

Evidence suggests a lack of confidence amongst FY2/SHO doctors in managing ophthalmic emergencies in the UK. Confidence is also low in performing funduscopy and using the slit lamp, and little formal ophthalmology training is received in the ED. Several of the available studies were of poor methodological quality, and evidence investigating the confidence levels of EM physicians was lacking. Further high-quality national surveys to investigate the confidence of EM physicians in managing eye emergencies are warranted. There is also a need to assess why foundation doctors feel ill-prepared to manage eye emergencies and to develop appropriate training to ensure good patient outcomes as the burden of ophthalmic presentations continues to grow.

## Summary

### What was known before

- Eye emergencies comprise a significant portion of attendances to general Emergency Departments (EDs) and the demand for emergency eye care is increasing.
- Previous studies have shown that FY2 doctors working in EDs often lack confidence in diagnosing and managing ophthalmic cases.

### What this study adds

- This is the first systematic review investigating confidence amongst doctors working in EDs in managing ophthalmic emergencies, performing funduscopy and using the slit-lamp.
- Confidence is lacking amongst foundation and SHO doctors, but there is insufficient and poor-quality evidence to draw conclusions about Emergency Medicine physicians.
- A majority of Emergency Medicine physicians are not confident in performing a lateral canthotomy and cantholysis for retrobulbar haemorrhage.
- Formal ophthalmology training provided by EDs is widely perceived as inadequate.

## Supplementary information

Summary of Supplementary Information

Supplementary Table 1

Supplementary Table 2

Supplementary Table 3

## Data Availability

All data generated and analysed are included in this published paper.
