# Supplementary material for: Confidence of Emergency Department doctors in managing ophthalmic emergencies: a systematic review
Source: Eye (Lond). 2024 May 10;38(14):2751–60. doi: 10.1038/s41433-024-03115-z (PMC11427453; doi:10.1038/s41433-024-03115-z)
Supplement: Supplementary file 1 — Summary of Supplementary Information [file 41433_2024_3115_MOESM1_ESM.docx]

**Summary of Supplementary Information**

**Confidence of Emergency Department doctors in managing ophthalmic emergencies: a systematic review**

Jessica Mendall, Abraham Tolley, Veronica Parisi, Stella Hornby, Ruth Brown & Victoria Nowak

**Supplementary Table 1: Complete search strategy for electronic databases.**

This table contains the complete search strategy of the systematic review, including details of the database, date of the last search, complete search strategy terms, and corresponding results of each search.

File type: Excel Workbook (.xlsx)

**Supplementary Table 2: Reasons for exclusion of full-text articles.**

This table includes all the full-text articles that were excluded during the full-text screening stage of the search, as mentioned in the PRISMA diagram (Figure 1). This table includes the author, year and title of the article, and the reason for exclusion.

File type: Excel Workbook (.xlsx)

**Supplementary Table 3: Risk of bias assessment using the Appraisal tool for Cross-Sectional Studies.**

This table provides the detailed results of the risk of bias assessment using the Appraisal tool for Cross-Sectional Studies (AXIS tool). It shows how each study scored for the individual components of the AXIS tool.

File type: Excel Workbook (.xlsx)
